# The Standard Preliminary to a Medical Education

**Published:** 1899-10

**Authors:** 


WHILE every effort is being made in this country to raise the standard preliminary to a medical education, the French have lowered theirs, and by a recent decree are to admit graduates from the so-called "modern" course—chemistry, physics and natural history—and no longer exact the diploma of the "classic" course—letters and philosophy. A similar movement has been agitated in Germany, but is frowned on in official circles, which even refuse to accept the classic diplomas of the Swiss Universities, on account of the fact that Greek is optional and may be substituted by a modern language.

